# Serum Resolvin D1 Levels as a Potential Trait Marker for Schizophrenia

**DOI:** 10.1192/j.eurpsy.2026.10629

**Published:** 2026-07-31

**Authors:** U. Ozkal

**Affiliations:** Department of Psychiatry, Sancaktepe Prof. Dr. İlhan Varank Training and Research Hospital, Istanbul, Türkiye

## Abstract

**Introduction:**

Schizophrenia is a severe neuropsychiatric disorder with emerging evidence implicating neuroinflammation and immune dysregulation in its pathophysiology. Resolvin D1 (RvD1), a specialized pro-resolving lipid mediator derived from omega-3 fatty acids, exhibits potent anti-inflammatory, anti-apoptotic, and neuroprotective properties. This study explores the role of RvD1 in schizophrenia, focusing on its potential as a trait marker.

**Objectives:**

This study aimed to compare serum RvD1 levels between patients diagnosed with schizophrenia using the Structured Clinical Interview for DSM-5 (SCID-5) and healthy controls, and to evaluate its suitability as a trait marker for the disorder.

**Methods:**

A total of 81 participants (55 patients with schizophrenia, 26 healthy controls) were included. Serum RvD1 levels were measured. Non-normal data distribution (Shapiro-Wilk test, p<0.05) led to the use of a Mann-Whitney U test for group comparison. Effect size was calculated using Cohen’s d.

**Results:**

Serum RvD1 levels were significantly elevated in schizophrenia patients compared to controls (p = 0.00119). The median RvD1 level was markedly higher in the schizophrenia group (457.54 pg/ml; IQR: 374.71–561.33) compared to the control group (373.52 pg/ml; IQR: 364.61–399.66). The effect size was moderate (Cohen’s d ≈ 0.49), indicating a clinically relevant difference.

**Conclusions:**

This study demonstrates significantly higher serum RvD1 levels in schizophrenia patients, suggesting a compensatory anti-inflammatory response to underlying neuroinflammation. These findings position RvD1 as a promising trait marker for schizophrenia. Further validation in larger cohorts and investigation into its mechanistic pathways are recommended to confirm its diagnostic and therapeutic potential.

**Disclosure of Interest:**

None Declared

